# Prevalence of Behavioral Health Conditions in a Safety-Net General Internal Medicine Practice

**DOI:** 10.1007/s11606-024-09264-x

**Published:** 2024-12-12

**Authors:** Roberto Lopez Cervera, Becky Ford, Tyler Winkelman

**Affiliations:** 1 University of Minnesota Medical School, Minneapolis, USA; 2 Health, Homelessness, and Criminal Justice Lab, Hennepin Healthcare Research Institute, Minneapolis, USA; 3 Research and Evaluation Data Analytics Core, Hennepin Healthcare Research Institute, Minneapolis, USA; 4 General Internal Medicine, Department of Medicine, Hennepin Healthcare, Minneapolis, USA

## INTRODUCTION

Over the last decade, the number of primary care visits related to mental health increased 50%.^1^ In addition to rising mental health needs among patients seeking primary care, there have also been well-reported increases in opioid use and opioid-related morbidity and mortality. One percent of primary care patients now have an opioid use disorder. ^2^ The prevalence of mental health conditions including substance use disorders, collectively referred to as behavioral health conditions, is even higher in safety-net settings.^3,4^

Seventeen percent of adults reported depression or anxiety symptoms in 2024, compared to 6–8% prior to the COVID-19 pandemic.^5^ However, the prevalence of behavioral health conditions in safety-net primary care settings is not well documented. These data are critical for informing practice and payment models to meet the shifting needs of primary care patients, particularly in safety-net settings that are typically resource-constrained.^6^ In this report, we explore the prevalence of behavioral health conditions in a large, urban general internal medicine safety-net practice by sex, race/ethnicity, and patient language. We also examine the overlap of these conditions with chronic physical health conditions given the impact of behavioral health conditions on management of physical health conditions.^7^

## METHODS

All analyses used de-identified data from patients 18 and older who visited the general internal medicine clinic at a safety-net health system in Minneapolis, MN, from January 1, 2021, to December 31, 2023. We compared patients with the most common behavioral health conditions, which included anxiety disorders, depressive disorders, alcohol use disorder, opioid use disorder, and/or stimulant use disorder to patients without these diagnoses. This combination of conditions was chosen because the clinic offers evidence-based medications for these conditions. Conditions were defined using International Classification of Diseases, Tenth Revision, Clinical Modification codes: F.32* (depressive disorders), F.41* (anxiety disorders), F.11* (opioid use disorders), F.10* (alcohol use disorders), F14* (cocaine use disorders), and F.15* (other stimulant use disorders). Among patients with behavioral health conditions, we compared demographic characteristics among people who were diagnosed with anxiety disorders and/or depressive disorders only (AD/DD), substance use disorder (SUD) only, or both (AD/DD) and SUD. We compared demographic characteristics across groups with chi-squared tests.

We used a de-identified dataset; therefore, our study was determined to not be human subjects research by the Hennepin Healthcare Research Institute Institutional Review Board.

## RESULTS

Our sample consisted of 72,499 patients ages 18 and older with at least one visit to a general internal medicine clinic over a 3-year period. Of these patients, 38.6% were diagnosed with at least one behavioral health condition, which suggests a substantial opportunity for integrated treatment in the safety-net primary care setting (Table 1). In particular, more than half of patients who identified as American Indian/Alaska Native (AI/AN), non-Hispanic White, or had one of the reported physical health conditions had a behavioral health condition. Patients who spoke a language other than English had a lower prevalence of behavioral health conditions compared to patients who spoke English.

**Table 1:** Prevalence of Behavioral Health Conditions by Patient Characteristics in a Safety-Net GIM Practice

Characteristic	Patients (row %)	
	Behavioral health condition	No behavioral health condition
Total	27,987 (38.6)	44,512 (61.4)
Mean age	46	34
Sex*		
Female	14,100 (39.8)	21,364 (60.2)
Male	13,884 (41.1)	23,145 (58.9)
Race/ethnicity*		
AI/AN	1372 (64.1)	769 (35.9)
Asian or Pacific Islander	773 (26.3)	2166 (73.7)
Black	10,749 (37.4)	18,011 (62.6)
Hispanic	4250 (25.4)	12,455 (74.6)
White, non-Hispanic	11,522 (52.3)	10,513 (47.7)
Language*		
English	24,644 (45.0)	30,134 (55.0)
Somali	394 (12.4)	2795 (87.6)
Spanish	2500 (21.1)	9334 (78.9)
Hmong	46 (28.8)	114 (71.2)
Other	403 (15.9)	2135 (84.1)
Physical health condition*		
Any	21,346 (56.4)	16,517 (43.6)
Diabetes mellitus, type II	5387 (52.9)	4798 (47.1)
Hypertension	10,978 (55.7)	8727 (44.3)
Heart failure	1992 (64.1)	1117 (35.9)
Ischemic heart disease	2989 (61.5)	1875 (38.5)
None	15,286 (31.0)	33,944 (69.0)

^*^ = *P* < 0.001

Among patients with behavioral health conditions, the proportion of patients with AD/DD only was 58.1%, SUD only was 13.7%, and co-occurring AD/DD and SUD was 28.2% (Table 2). Specifically, substance use was more common among male patients and patients with chronic physical health conditions. Types of behavioral health conditions had substantial variation across demographic groups. Notably, over half of patients who identified as AI/AN were diagnosed with both AD/DD and SUD.

**Table 2:** Prevalence of Anxiety Disorders/Depressive Disorders, Substance Use Disorders, or Both Among Patients with Behavioral Health Conditions in a Safety-Net GIM Practice

Characteristic	Patients (row %)		
	Anxiety disorder and/or depressive disorder (AD/DD) only	Ssubstance use disorder (SUD) only	AD/DD + SUD
Total	16,273 (58.1)	3833 (13.7)	7881 (28.2)
Mean age	43	51	49
Sex*			
Female	10,131 (71.8)	885 (6.3)	3084 (21.9)
Male	6140 (44.2)	2947 (21.2)	4797 (34.6)
Race/ethnicity*			
AI/AN	396 (28.9)	269 (19.6)	707 (51.5)
Asian or Pacific Islander	551 (71.3)	78 (10.1)	144 (18.6)
Black	5561 (51.7)	1802 (16.8)	3386 (31.5)
Hispanic	3412 (80.3)	353 (8.3)	485 (11.4)
White, non-Hispanic	6543 (56.8)	1430 (12.4)	3549 (30.8)
Language*			
English	13,377 (54.3)	3571 (14.5)	7696 (31.2)
Somali	325 (82.5)	34 (8.6)	35 (8.9)
Spanish	2193 (87.7)	190 (7.6)	117 (4.7)
Hmong	44 (95.7)	–	–
Other	334 (82.9)	36 (8.9)	33 (8.2)
Physical health condition*			
Any	10,487 (49.1)	3438 (16.1)	7421 (34.8)
Diabetes mellitus, type II	2893 (53.7)	803 (14.9)	1691 (31.4)
Hypertension	5439 (49.5)	1753 (16.0)	3786 (34.5)
Heart failure	821 (41.2)	363 (18.2)	808 (40.6)
Ischemic heart disease	1334 (44.6)	519 (17.4)	1136 (38.0)
None	9947 (65.1)	1760 (11.5)	3579 (23.4)

^*^ = *P* < 0.001

– = small sample size

## CONCLUSIONS

Within a general internal medicine clinic in a Midwestern safety-net health system, nearly two in five patients were diagnosed with a behavioral health condition. Patients who identified as AI/AN or White or were diagnosed with a chronic physical health condition had a particularly high prevalence of behavioral health conditions. These estimates highlight that safety-net clinics are an important front door for patients needing behavioral health treatment. The high prevalence of substance use disorders and co-occurring disorders among patients who identified as Black or AI/AN also highlights the need for culturally specific approaches to treatment.

Models that integrate behavioral health services into primary care, like the Collaborative Care Model, could improve the sustainability of primary care practices that serve patients with a high burden of behavioral and physical health conditions. The future of primary care and behavioral health care in the USA are intertwined and reforms that target both should be prioritized.
